# Structural integrity of additively manufactured materials

**DOI:** 10.1038/s41598-025-21342-6

**Published:** 2025-10-03

**Authors:** Thomas Niendorf, Sara Bagherifard, Andrey Molotnikov, Giovanni Bruno

**Affiliations:** 1 Institute of Materials Engineering, University of Kassel, Moenchebergstr. 3, 34125 Kassel, Germany; 2 Department of Mechanical Engineering, Politecnico di Milano, Via La Masa, 1, 20156 Milan, Italy; 3 RMIT Centre for Additive Manufacturing, RMIT University, 124 La Trobe Street, Melbourne, VIC 3000 Australia; 4 BAM, Bundesanstalt für Materialforschung und -prüfung, Unter den Eichen 87, 12205 Berlin, Germany; 5 Institute of Physics and Astronomy, University of Potsdam, Karl-Liebknecht-Str. 24-25, Potsdam, Germany

**Keywords:** Materials science, Structural materials

Additive manufacturing (AM) enables the direct conversion of complex shapes obtained from computer-aided design or 3D scanning data into physical objects. Thanks to its digital flexibility and efficiency, AM offers unprecedented solutions in the design and production of complex structural components. Such components can even be at the same time multi-functional, topologically optimized, easier to produce, and more cost-effective. On the other hand, extending these benefits to large-scale and load-bearing industrial applications requires a deeper understanding of the mechanical behaviour of these materials. How the microstructure of AM materials, process-related attributes, surface conditions, and distribution of internal defects influence the mechanical behaviour in fact remains a major open area of research.

The Collection on “Structural integrity of AM materials” brings together research articles advancing our current understanding of the interplay between microstructures and process attributes, in order to determine the structural integrity of AM materials. Papers were collected and chosen to address all important current activities in the field including fatigue and fracture, characterization, testing, and modelling of AM components, as well as process monitoring and novel structural integrity assessment techniques. These are grouped as follows:

In recent years advanced process monitoring and characterization techniques, the latter making use of X-ray, synchrotron and neutron diffraction, have been applied and optimized to provide for new insights into the evolution of defects and the unique characteristics of AM processed materials and components. The high intensity and brilliance of some of these facilities allows probing AM materials not only with high spatial resolution inside a given volume, but also with unprecedented temporal resolution. Consequently, operando and in situ studies, respectively, are used to shed light on local microstructure and defect evolution even in the case of the extremely rapid AM processes. Three of the papers included in the Collection report on recent progress in application of process monitoring and advanced characterization techniques: “Defect detection by multi-axis infrared process monitoring of laser beam directed energy deposition”, “Understanding the hot isostatic pressing effectiveness of laser powder bed fusion Ti–6Al–4V by in-situ X-ray imaging and diffraction experiments” and “In situ observation of melt pool evolution in ultrasonic vibration-assisted directed energy deposition”.

Still, there is an urgent need for the application of post treatments to finally tailor the microstructure and properties of AM materials. For superior structural integrity the post treatments applied have to follow the initial AM process in an adequate way such that a wide variety of treatments (thermal, mechanical or thermo-mechanical) have to be considered. The Collection addresses comprehensively the effectiveness and optimization potential of different post treatments in the following three papers: “Accelerated annealing of fused filament fabricated (FFF) thermoplastics via an improved core–shell filament”, “Mechanical properties of additively manufactured zirconia with alumina air abrasion surface treatment” and “The efficiency of tumble finishing as a final post-treatment for fatigue enhancement of notched laser powder bed fusion AlSi10Mg”.

To further elaborate in-depth knowledge on most important influencing factors for the assessment of structural integrity of AM materials and components, efficient and reliable models are required. These models include defect-based prediction tools for assessment of fatigue lives as well as approaches looking at crystal plasticity for understanding of the role of local microstructure, including heterogeneities of any kind, on the overall material behavior. Three papers are included in the Collection reporting on advances in the field of modeling and simulation: “Strain-based method for fatigue failure prediction of additively manufactured lattice structures”, “Crystal plasticity simulations with representative volume element of as-build laser powder bed fusion materials” and “Experimental scatter of the fatigue response of additively manufactured components: a statistical method based on the Profile Likelihood”.

Finally, the Collection contains four papers focusing on specific properties of aluminum alloys and advanced high-strength steels, closely looking at process-microstructure-damage evolution relationships. In these papers synergistic characterization techniques are used to elaborate a comprehensive understanding to eventually substantiate promising pathways toward the development of novel alloys being characterized by superior damage tolerance and, thus, unprecedented structural integrity: “Enhanced structural integrity of Laser Powder Bed Fusion based AlSi10Mg parts by attaining defect free melt pool formations”, “The role of internal defects on anisotropic tensile failure of L-PBF AlSi10Mg alloys”, “Effect of processing parameters on texture and variant selection of as-built 300 maraging steel processed by laser powder bed fusion” and “Metastable CrMnNi steels processed by laser powder bed fusion: experimental assessment of elementary mechanisms contributing to microstructure, properties and residual stress”.

We trust that this Collections stimulates further advances in AM and paves the way for widespread use of AM technologies in any kind of industrial field. We are convinced that all readers will receive inspiration from the results presented to guide their own research.

On behalf of all guest authors, 

Sara Bagherifard – Politecnico di Milano, Italy

Giovanni Bruno – BAM, Bundesanstalt für Materialforschung und -prüfung, Berlin, and University of Potsdam, Germany

Andrey Molotnikov – RMIT University, RMIT Centre for Additive Manufacturing, Australia

Thomas Niendorf – University of Kassel, Germany

